# A Review of Quality Measures for Assessing the Impact of Antimicrobial Stewardship Programs in Hospitals

**DOI:** 10.3390/antibiotics5010005

**Published:** 2016-01-13

**Authors:** Mary Richard Akpan, Raheelah Ahmad, Nada Atef Shebl, Diane Ashiru-Oredope

**Affiliations:** 1 Department of Pharmacy, Pharmacology and Postgraduate Medicine, University of Hertfordshire, Hatfield, AL10 9AB, UK; m.akpan@herts.ac.uk (M.R.A.); n.a.shebl@herts.ac.uk (N.A.S.); 2 NIHR Health Protection Research Unit in Healthcare Associated Infection and Antimicrobial Resistance at Imperial College London, Hammersmith Campus, Du Cane Road, London, W12 0NN, UK; raheelah.ahmad00@imperial.ac.uk; 3 Antimicrobial Resistance Program, Public Health England, London, NW9 5EQ, UK

**Keywords:** antimicrobial stewardship, antimicrobial resistance, quality indicators, outcome, patient, infectious diseases

## Abstract

The growing problem of antimicrobial resistance (AMR) has led to calls for antimicrobial stewardship programs (ASP) to control antibiotic use in healthcare settings. Key strategies include prospective audit with feedback and intervention, and formulary restriction and preauthorization. Education, guidelines, clinical pathways, de-escalation, and intravenous to oral conversion are also part of some programs. Impact and quality of ASP can be assessed using process or outcome measures. Outcome measures are categorized as microbiological, patient or financial outcomes. The objective of this review was to provide an overview of quality measures for assessing ASP and the reported impact of ASP in peer-reviewed studies, focusing particularly on patient outcomes. A literature search of papers published in English between 1990 and June 2015 was conducted in five databases using a combination of search terms. Primary studies of any design were included. A total of 63 studies were included in this review. Four studies defined quality metrics for evaluating ASP. Twenty-one studies assessed the impact of ASP on antimicrobial utilization and cost, 25 studies evaluated impact on resistance patterns and/or rate of *Clostridium difficile* infection (CDI). Thirteen studies assessed impact on patient outcomes including mortality, length of stay (LOS) and readmission rates. Six of these 13 studies reported non-significant difference in mortality between pre- and post-ASP intervention, and five reported reductions in mortality rate. On LOS, six studies reported shorter LOS post intervention; a significant reduction was reported in one of these studies. Of note, this latter study reported significantly (*p* < 0.001) higher unplanned readmissions related to infections post-ASP. Patient outcomes need to be a key component of ASP evaluation. The choice of metrics is influenced by data and resource availability. Controlling for confounders must be considered in the design of evaluation studies to adequately capture the impact of ASP and it is important for unintended consequences to be considered. This review provides a starting point toward compiling standard outcome metrics for assessing ASP.

## 1. Introduction

Antimicrobial resistance (AMR) is a growing public health threat which has attracted the attention of national and international bodies. A recent World Health Organization (WHO) surveillance of resistance to antibacterial drugs in bacteria commonly associated with hospital and community infections revealed increasing resistance and/or decreased susceptibilities in the studied bacteria [1]. Resistance of *Escherichia coli* to third-generation cephalosporins and fluoroquinolones and *Staphylococcocus aureus* to methicillin (Methicillin Resistant *Staphylococcous aureus*, MRSA) are reported to be 50% or more in five out of the six WHO regions [1]. *Klebsiella pneumoniae* resistance to third-generation cephalosporins is reported to be greater than 50% in all six WHO regions. Carbapenem-resistant *K. pneumoniae* is reportedin all WHO regions, with reports in two regions exceeding 50%. Also, non-susceptibility of *Streptococcus pneumoniae* to penicillin is reported to be more than 50% in all six WHO regions. A related English AMR surveillance report revealed increased resistance of *E. coli* and *K*. *pneumoniae* to ciprofloxacin, third-generation cephalosporins, gentamicin, and imipenem/meropenem [2]. The report however indicated decreased resistance of *Pseudomonas aeruginosa* to ceftazidime, gentamicin, and imipenem/meropenem [2]. Recent reports highlightthat patients with infection caused by drug resistant bacteria have a two-fold increase in mortality compared to those with infection with sensitive bacteria [1,3].

Available estimates indicate that between 25%–50% of hospitalized patients receive antibiotics, with between 30% and 50% of antibiotic use being inappropriate [4,5]. Published literature demonstrates a strong link between antibiotic use and the development of resistance [3,6,7,8]. Antimicrobial stewardship programs are therefore quality motivated interventions aimed at improving the use of antibiotics in healthcare facilities. The primary goal is to optimize clinical outcomes and minimize unintended consequences such as *Clostridium difficile* infection (CDI) and resistance [4,9]. Strategies to achieve these goals have included prospective audit with intervention and feedback, and formulary restriction and preauthorization. Supplemental strategies include education, guidelines and clinical pathways, antimicrobial cycling and scheduled antimicrobial switch, antimicrobial order forms, automatic stop orders, combination therapy, streamlining or de-escalation of therapy, dose optimization, conversion from parenteral to oral therapy, and computer surveillance and decision support [4,10].

A Cochrane review of interventions to improve antibiotic prescribing for hospital inpatients classified these strategies into three main groups namely:

- persuasive interventions: these include education, audit and feedback, guidelines and clinical pathways.
- restrictive interventions: formulary restriction, prior approval or preauthorization from infectious diseases (ID) physician, microbiologist or pharmacists, automatic stop orders, antimicrobial cycling or scheduled switch, antibiotic order forms.
- structural interventions: computerized records, computerized decision support, example computer physician order entry (CPOE) [11].

Effective antimicrobial stewardship programs require a multidisciplinary team with responsibility for promoting prudent antimicrobial use. The Infectious Diseases Society of America and the Society for Healthcare Epidemiology of America (IDSA/SHEA) ASP guidelines [4] recommend a multidisciplinary team which includes an ID physician and a clinical pharmacist with infectious diseases training as core members. Inclusion of a clinical microbiologist, information system specialist, infection control specialist, hospital epidemiologist, and hospital administrator is considered optimal. The English antimicrobial stewardship (AMS) toolkit: “Start Smart, then Focus” recommends other core members should be present, an acute care physician, a surgeon, a senior member of the pharmacy management team, an anesthetist, a pediatrician, and a senior nurse [12].

There is an increased call on healthcare organizations to develop quality measures or indicators to monitor and evaluate the impact of ASP [4,12,13,14]. Previous reviews have reported on the impact of ASP in reducing antimicrobial cost, AMR, superinfection, and patient outcomes (such as length of stay (LOS), readmission rate, and mortality) [11,15]. The objective of this review was to provide an overview of reported quality measures for assessing ASP and report on the impact of published antimicrobial stewardship studies on these measures, with particular focus on patient outcome measures.

## 2. Methods

A literature search of papers published in English between the 1990 and June 2015 was identified through a search of five databases: Scopus, Medline, CINAHL, Pubmed, and Embase using the following search terms: (“antimicrobial stewardship” OR “antimicrobial stewardship program” OR “antibiotic control program” OR “antibiotic policy” OR “antibiotic management program”); (outcomes OR impact OR “quality measure” OR “performance measures” OR “length of stay” OR “clinical improvement” OR “*C. difficile* infection” OR mortality OR resistance OR readmission OR MRSA).

### Study Inclusion and Exclusion Criteria

Only primary research studies, published in English and which met the following criteria were included:

- Defined and/or developed quality measures for assessing ASP in hospital settings.
- Used quality performance measures (such as change in antimicrobial use) and outcome measures (including resistance patterns, rates of CDI, LOS, readmission, mortality, and cost savings) in evaluating impact of ASP.
- Involved adult inpatients in acute and community hospital settings.

Studies excluded were:

- Those that reported prevalence of ASP without evaluation of impact.
- ASP studies in pediatrics and long term care facilities.

## 3. Results

The initial search returned 4319 articles. Of these, 152 met the inclusion criteria, and a full-text evaluation was carried out on 63 studies. In summary, four studies defined quality metrics for evaluating ASP. Twenty-one studies assessed impact on antimicrobial utilization and cost, 25 studies evaluated impact on resistance patterns and/or rate of CDI. Thirteen studies assessed impact on patient outcomes including mortality, LOS, and readmission rates.

### 3.1. Studies that Defined Quality Measures for Evaluating ASP

Table 1 includes studies [16,17,18,19] that developed or defined quality measures for evaluating ASP. Methods used were modified Delphi technique, survey, and interviews. Bumpass *et al*. [19] surveyed ID physicians’ and pharmacists’ opinions of AMS metrics considered important in evaluating ASP. The authors reported that although appropriateness of antimicrobial use, infection-related mortality, and antibiotic associated length of stay were considered more important outcomes by those surveyed, antimicrobial use and cost were the most commonly collected metrics.

**Table 1:** Studies that defined quality measures for evaluating antimicrobial stewardship programs antimicrobial stewardship programs (ASP).

Study	Method Used	Category of Measure	Quality Measures Identified
Nathwani *et al*., 2002 [16]	Expert panel	Process measures for glycopeptideprescribing	total number of glycopeptide in defined daily dose (DDD)/1000-patient days number of alert antibiotic forms completed for glycopeptide number of patients prescribed glycopeptide appropriately according to policy number of patients prescribed glycopeptide inappropriately
Chen *et al*., 2011 [17]	Survey by questionnaireand interviews	Process and outcome	DDD/1000 patient-days against state or national data quantity of antimicrobial use within hospital number of prescriptions of restricted antibiotic complaints with approved guideline. cost savings
Morris *et al*., 2012 [18]	Modified Delphi	Process and outcome	days of therapy/1000 patient-days number of patients with specific organisms that are drug resistant mortality related to antimicrobial-resistant organisms conservable days of therapy among patients with community-acquired pneumonia (CAP), skin and soft-tissue infections (SSTI), or sepsis and bloodstream infections (BSI) unplanned hospital readmission within 30 days after discharge from the hospital in which the most responsible diagnosis was one of CAP, SSTI, sepsis or BSI
Bumpass *et al*., 2014 [19]	Survey	Process and outcome	appropriateness of antimicrobial use infection-related mortality rate antibiotic-associated length of stay antimicrobial use antimicrobial cost

DDD—Defined daily dose.

### 3.2. Impact of ASP on Quality Measures

Impact of ASP on different quality measures is summarized in Table 2, Table 3 and Table 4. This is grouped into:

- impact on antimicrobial use and cost savings
- impact on *C. difficile* infection and resistance patterns
- impact on patient outcomes (LOS, readmission rate, mortality)

Some of the studies used more than one measure in assessing impact and majority (29) employed before-after or pre-post-intervention (quasi-experimental) design without control. The pre-phase consisted of retrospective collection of baseline data before ASP implementation.

#### 3.2.1. Impact of ASP on Antimicrobial Use and Cost of Antimicrobials

Change in the use of specific antibiotic or antibiotic class is considered a process measure [4,20]. The majority of the programs that assessed impact of ASP on antibiotic use also assessed cost savings. Twenty-one studies assessed impact on antimicrobial use and/or cost. The majority of the studies that reported significant cost savings did not provide the cost of implementing the program. Table 2 summarizes studies [21,22,23,24,25,26,27,28,29,30,31,32,33,34,35,36,37,38,39,40,41] that assessed the impact of ASP on antimicrobial use and cost of antimicrobials.

**Table 2:** Impact of ASP on antimicrobial use and cost of antimicrobials.

Study	Setting	AMS Strategy	Design	Results
Mercer *et al*., 1999, USA [21]	450-bed community hospital	Restriction, pre-authorization, clinical pathway	Before-after	Cost of IV and oral antibiotics reduced by 26% and 10% respectively. Use of high cost IV antibiotics reduced by 22%.
Bassetti *et al*., 2000, Italy [22]	2500-bed teaching hospital	Formulary restriction, sequential therapy.	Before-after	Cost of antibiotics decreased by 10.5% following formulary introduction with cost savings of €345,000. Ceftazidime cost reduced by 52%, and antibiotic cost per day of hospital stay decreased from €4.53 to €4.18.
Berlid *et al*. 2001, Norway [23]	600-bed acute hospital	Guidelines, education	Prospective before-after	23% reduction in use of broad-spectrum antibiotics. Cost of antibiotic reduced by 27% and 32% in the first and second year of the program respectively.
Ansari *et al*. 2003, Scotland, UK [24]	900-bed University-affiliated hospital	Guideline, review and feedback	Before-after with interrupted time series (ITS)	Cost savings from targeted antibiotics was (£133,269) (*p* < 0.0001). Cost of program was £20,133. Use of targeted antibiotic reduced by 0.27 DDD/100 bed-days/month.
Cook *et al*., 2004, USA [25]	730-bed university teaching hospital	Restriction, pre-authorization, review and feedback	Before-after	Broad spectrum antibiotic use decreased by 28% with no change in susceptibilities of common nosocomial gram-negative organisms.
Mcgregor *et al*., 2006, USA [26]	648-bed, tertiary-care referral center	Computerized decision support system, review and feedback	Randomized controlled trial	Cost savings of $84,194 (23%) in the intervention group.
Siddiqui *et al*., 2007, Pakistan [27]	12-bed adult ICU at a teaching hospital	Restriction policy, stamp on chart, feedback	Before- after	34% reduction in use of broad spectrum antibiotics and 40% cost reduction.
Cheng *et al*. 2009, Hong Kong [28]	1500-bed university- affiliated hospital	Guidelines, education, feedback	Before-after	Antimicrobial use reduced from 73.06 (baseline) to 64.01 DDD/1000 patient-days.Reduction in broad-spectrum intravenous and total antibiotics expenditure.
Teo *et al*., 2012, Singapore [29]	1700-bed teaching hospital	Guidelines, algorithm, review, audit and feedback,	Before-after	9.9% decrease in antibiotic consumption (*p* = 0.032) with cost savings of $198,575 for the hospital, and $91,194 for patients.
Michaels *et al*., 2012, USA [30]	236-bed acute-care community hospital	Restriction, review and feedback, guidelines, education	Before-after	Antimicrobial use decreased from 821.33 DDD/1000 patient-days to 778.77 DDD/1000 patient-days. Cost savings approached $290,000 from reduction in antibiotic expenditure.
Hagert *et al*., 2012, USA [31]	39-bed acute care and 38-bed community hospital	Computerized decision support system, review and feedback	Retrospective (before-after) chart review	Percentage of patients on antimicrobial decreased from 36.8% to 25% (*p* < 0.001). Total inpatient antimicrobial costs decreased by $48,044
Vettese *et al*., 2013, USA [32]	253-bed Community hospital	IV to oral conversion, dose optimization, review	Before-after	6.4% decline in days of therapy and a 37% reduction in total antimicrobial expenditure.
Cisneros *et al*., 2014, Spain [33]	1251-bed teaching hospital	Education and training, guidelines, counseling interviews, feedback	Before-after	Reduction in antimicrobial consumption from 1150 DDD/1000 patient-days to 852 DDD/1000 patient-days with 42% reduction in antimicrobial expenditure.
Borde *et al*., 2014, Germany [34]	1600-bed teaching hospital	Guidelines revision information and education, review and feedback	Before-after with interrupted time series	Significant decline in overall antibiotic use (*p* < 0.0001), significant decrease in cephalosporins and fluoroquinolones use (*p* < 0.001).
Bartlett & Siola, 2014, USA [35]	155-bed community hospital	Formulary restriction, IV to oral conversion, automatic stop, review and feedback	Before-after	Acquisition costs decreased by 25.5%, from $569,786 to $424,433 with a direct cost savings of $145,353. Antimicrobial use decreased from 1627 to 1338 DDD/1000 patient-days, a decrease of 17.8%.
Hou *et al*., 2014, China [36]	12-bed ICU of a 700-bed tertiary teaching hospital	Education, formulary restriction & preauthorization	Before-after	Total ICU antibiotic consumption decreased from 197.65 to 143.41DDD/100patient-days with improvement in bacterial resistance. Hospital-wide consumption also decreased from 69.69 DDDs to 50.76 (27.16% decrease)
Palmay *et al*., 2014, Canada [37]	6 clinical service sections at a 1275-bed university hospital	Education, audit and feedback	Stepped-wedge randomized trial	ASP intervention was associated with 21% reduction in targeted antimicrobial (*p* = 0.004) with no reduction in cost and microbiological outcomes.
Chandy *et al*., 2014, India [38]	2140-bed teaching hospital	Antibiotic policy guidelines	Segmented time series	Overall antibiotic use increased at a monthly rate in segments 1, 2 & 3 of the study but drop significantly in monthly antibiotic use in segment 5.
Fukuda *et al.*, 2014, Japan [39]	429-bed community hospital	Prospective audit with intervention and feedback, dose optimization, de-escalation	Before-after	25.8% decrease in antimicrobial cost (*p* = 0.005), 80.0% decrease in aminoglycosides use (*p* < 0.001).
Cook & Gooch, 2015, USA [40]	904-bed, tertiary-care teaching hospital	Restriction and prior approval, review and feedback, automatic stop	Prospective interventional	Total antimicrobial use decreased by 62.8% (*p* < 0.0001). Aminoglycosides use decreased by 91.3% (*p* < 0.0001), cephalosporins decreased by 68.3% (*p* < 0.0001), extended-spectrum penicillins decreased by 77.7% (*p* < 0.0001), quinolones by 78.7% (*p* < 0.0001). Antifungal use decreased by 71.0% (*p* < 0.0001) during 13-year study period.
Taggart *et al*., 2015, Canada [41]	2 ICUs at a 465-bed teaching hospital	Audit and feedback	Controlled before-after withinterrupted time series	Total monthly antimicrobial use in one of the ICUs decreased by 375 DDD/1000 patient-days (*p* < 0.0009) after intervention.

#### 3.2.2. Impact of ASP on Resistance Patterns and Clostridium Difficile Infection (CDI)

A total of 25 studies [42,43,44,45,46,47,48,49,50,51,52,53,54,55,56,57,58,59,60,61,62,63,64,65,66] assessed the impact of ASP on microbiological outcomes (bacterial resistance patterns) and/or CDI rates. Thirteen of the 25 studies assessed impact on CDI rates and other outcomes. Nine out of 13 studies reported a statistically significant reduction in the rate of CDI following ASP implementation [41,42,51,52,54,56,58,61,64]. Khan and Cheesbrough [44] and Malani *et al*. [62] reported a progressive fall in the rate of *C. difficile*-associated diarrhea (CDAD) and 50% decrease in the likelihood of developing CDI respectively. A restriction policy on ciprofloxacin and ceftriaxone resulted in a 70.20% reduction in the CDI with non-significant effect on extended spectrum beta lactamases (ESBL)—producing colliforms (*p* = 0.075) [63]—a proxy for antimicrobial resistance development.

Twelve studies assessed the impact on the resistance patterns of organisms commonly associated with hospital infections (ESKAPE: *Enterococcus faecium*, *Staphylococcocus aureus*, *Klebsiella pneumoniae*, *Acinetobacter baumannii*, *Pseudomonas aeruginosa*, and *Enterobacter* species, e.g., *Escherichia coli*) [67] and reported a reduction in resistance or unchanged susceptibilities in these organisms following AMS interventions. Saizy-Callaert *et al*. [45] reported a significant fall in the rate of ESBL-producing *Enterobacteriaceae* (*p* < 0.001) following the development of a multidisciplinary consultative approach which included developing local prescribing consensus with all prescribers; restricted prescriptions policy; regular audits of use of restricted antibiotics and institutional wide training and information for prescribers. A restriction policy on ceftazidime resulted in a significant decrease in *A. baumannii* (*p* = 0.01) [48]. Table 3 summarizes studies that assessed impact on CDI rate and resistance patterns.

**Table 3:** Impact of ASP on resistance patterns and *C. difficile* infection.

Study	Setting	AMS Strategy	Design	Results
McNulty *et al*., 1997, UK [42]	Elderly unit at a600-bed district hospital	Guideline, restriction following outbreak of CDI	Before-after	CDAD cases fell from 37 to 16 following restriction of cefuroxime.
Carling *et a*l., 2003, USA [43]	University-affiliated teaching hospital	Formulary, Prospectivemonitoring	Prospective interventional	Significant fall in rates of CDI and *Enterobacteriaceae* infections, (*p* = 0.002 and *p* = 0.02) respectively during 7 years of ASP.
Khan & Chessbrough 2003, UK [44]	800-bed district hospital	Formulary change, IV to oral conversion	Before-after	Progressive fall in incidence of CDAD over 5-year period.
Saizy-Callaert *et al*., 2003, France [45]	600-bed hospital with 5 teaching department	Guideline, restriction, training, feedback	Before-after	Significant fall in ESBL-producing *Enterobacteriaceae* (*p* < 0.001). MRSA and CRP rates remained stable.
Bantar *et al*., 2003, Argentina [46]	250-bed teaching hospital for adults	Antibiotic order form, feedback, education, prescription change	Prospective interventional	NS change in resistance of *E. coli* and *K. pneumoniae* to 3^rd^-generation cephalosporins, but decreasing resistance of *P. mirabilis* and *E. cloacae o*bserved. Imipenem-resistant *P. aeruginosa* decreased to 0%.
Martin *et al*., 2005, USA [47]	University hospital	Guidelines, formulary restriction	Prospective interventional	Increased susceptibility of *P. aeruginosa* to piperacillin/tazobactam, ceftazidime and fluoroquinolones. 3% reduction in MRSA rate and decreased resistance of *K. pnuemoniae* to ceftazidime.
Brahmi *et al*., 2006, Tunisia [48]	12-bed ICU	Ceftazidime restriction	Before-after	Significant (*p* = 0.01) decrease in *A. baumannii* to ceftazidime. Considerable reduction in ESBL-producing *K. pneumoniae*resistance to ceftazidime.
Ntagiopoulos *et al*., 2007, Greece [49]	12-bed ICU of 700-bed university-affiliated general hospital	Restriction of fluoroquinolones and ceftazidime	Before-after	Significant increase in susceptibilities of *A. baumannii, P. aeruginosa* and *K. pneumoniae* to ciprofloxacin (*p* < 0.01).
Mach *et al*., 2007, Czech Republic [50]	500-bed general hospital	Guidelines, restriction, education	Before-after	NS decrease in resistance to restricted antimicrobials, and NS increase in resistance to non-restricted antimicrobials. Decreased resistance of *E. aerogenes*and *K. pneumoniae* to ofloxacin, gentamicin and ceftazidime.
Fowler *et al*., 2007, UK [51]	Three acute-care wards for elder at a 1200-bed tertiary hospital	Narrow-spectrum’ antibiotic policy, feedback, cephalosporin restriction	Before-after with ITS	Significant (*p* = 0.009) fall in CDI, no reported rise in infection control procedures; MRSA remained unchanged (*p* = 0.32).
Valiquet *et al*.,2007, Canada [52]	683-bed secondary/tertiary care hospital	Guidelines, education	Before-after with ITS	Significant (*p* = 0.007) fall in CDAD incidence, no change (*p* = 0.63) following enhanced infection control.
Ozorowski *et al*., 2009, Poland [53]	120-bed hematology and blood transfusion tertiary care center	Guidelines, education	Before-after	Successful control of VRE outbreak and improvement in the resistance patterns of gram-negative bacteria.
Talpaert *et al*., 2011, [54]	450-bed university affiliated general hospital	Guideline and restriction of ‘high-risk’ antibiotics, education	Quasi-experimental with ITS	Significant fall in CDI incidence (*p* < 0.0001).
Altunsoy *et al*., 2011, Turkey [55]		Nation-wide restriction program	Before-after	Decrease in MRSA rates from 44% to 41%. Decrease in the use of carbapenems correlated with decrease in carbapenem-resistant *Pseudomonas* and *Acinetobacter* species.
Cook *et al*., 2011 [56]	861-bed university teaching hospital	EMR implementation	Before-after with ITS	18.7% decrease in CDI (*p* = 0.07) and 45.2% decrease in MRSA (*p* < 0.0001).
Niwa *et al*., 2012, Japan [57]	606-bed university hospital	Prospective review, guidelines, de-escalation, education	Before-after	Significant reduction in MRSA and *Serratia marcescens* occurrence (*p* = 0.026 and *p* = 0.026) respectively. NS decrease in *P. aureginosa* resistant to ceftazidime and piperacillin.
Aldeyab *et al*., 2012, UK [58]	233-bed hospital	Revised antibiotic policy that avoided ‘high-risk’ antibiotics	Retrospective intervention with ITS	Significant decrease in CDI incidence rate (*p* = 0.0081); CDI decreased by 0.0047/100 bed-days per month.
Jaggi *et al*., 2012, India [59]	Tertiary care hospital	Antibiotic policy, restriction, audit and feedback	Prospective interventional	4.03% reduction in carbapenem-resistant *Pseudomonas*. Rising trend in *E.coli*, *K. pnemoniae* and *A. baumannii*carbapenem resistance was recorded.
Sarraf-Yazdi *et al*., 2012, USA [60]	16-bed surgical ICU at an academic medical center	Antibiotic cycling	Controlled before-after	Improved susceptibility of pseudomonal isolates to ceftazidime (*p* = 0.003) and (piperacillin/tazobactam *p* = 0.02). Improved susceptibility of *E. coli* to piper/tazobactam (*p* < 0.0005).
Nowak *et al*., 2012, USA [61]	583-bed tertiary referral hospital	Computer surveillance & decision support system (data-mining software), education	Prospective before-after	Significant decrease in rates of CDI and VRE, (*p* = 0.018 and = 0.0004 respectively). NS difference in rate of MRSA (*p* = 0.09).
Malani *et al*., 2013, USA [62]	535-bed non-university affiliated community teaching hospital	Review, feedback, automatic stop, de-escalation	Retrospective observational	Likelihood of developing CDI decreased by 50% (*p* < 0.01).
Dancer *et al*, 2013, UK [63]	450-bed district general hospital	Education, restriction following outbreak	Prospective interventional	77% reduction in CDI rate. NS effect on MRSA rate (*p* = 0.62) and borderline effect of ESBL-producing coliforms (*p* = 0.075).
Wenisch *et al*., 2014, Austria [64]	1000-bed tertiary care community hospital	Moxifloxacin restriction, education	Before-after	46% reduction in CDI cases (*p* = 0.0044).
Knudsen & Andersen, 2014, Denmark [65]	University hospital	Guidelines, education	Controlled before-after with ITS	Significant reduction in ESBL-producing *K. pneumoniae* infections (*p* < 0.001).Significant increase in pipercillin-tazobactam-resistant *P. aeruginosa* and *E. faecium*infections were also recorded (*p* < 0.033).
Sarma *et al*., 2015, UK [66]	2 acute hospitals (combined bed 800)	Fluoroquinolone restriction	Before-after with ITS	Significant fall in CDI over a 60-month period.

NS—Non-significant, CRP—Ceftazidimie-resistant *Pseudomonas*, CDAD—*Clostridium difficile* associated diarrhea, ID—Infectious diseases, MRSA—Methicillin-resistant *Staphylococcus aureus*, CDI—*Clostridium difficile* infection, ESBL—Extended spectrum-producing beta-lactamases, VRE—Vancomycin-resistant enterococcus, SSI—surgical site infections, EMR—Electronic medical record.

#### 3.2.3. Impact of ASP on Patient Outcomes

Thirteen studies [68,69,70,71,72,73,74,75,76,77,78,79,80] were identified that reported impact on patient outcomes. Gums *et al*. [68] reported shorter LOS in the intervention group than the control group (9.0 *vs*. 5.7; *p* = 0.0001) and 6.3% mortality in the intervention group compare to 12.0% in the control group. Similarly, Ng *et al*. [70] reported a significant (7.46 and 6.97 respectively; *p* < 0.001) difference in LOS between the periods before and after ASP implementation with no difference in mortality. Six studies reported non-significant difference in mortality [69,71,72,73,75,78].Okumura *et al*. [80] however reported lower 30-day mortality with bundled ASP (intervention consisting of clinical pharmacist chart review, discussion with microbiologist and infectious disease physicians, local education and continuous follow-up) (*p* < 0.01) than conventional ASP. One study assessed incidence of adverse reactions following carbapenem de-escalation and reported that the de-escalated group had fewer adverse reaction (11/204 (5.4%) *vs.* 12/96 (12.5%); *p* = 0.037) [79].

A summary of studies that assessed impact on patient outcomes is provided in Table 4.

**Table 4:** Impact of ASP on patient outcomes.

Study	Setting	AMS Strategy	Design	Results
Gum *et al*., 1999, USA [68]	275-bed community hospital	Prospective review with intervention	Prospective RCT	Shorter LOS in the intervention group than the control group (9.0 *vs*. 5.7; *p* = 0.0001). Mortality rate was 12.0% (15/125) in the control group and 6.3% (8/127) in the intervention group.
Chang *et al*., 2006, Taiwan [69]	921-bed medical center	Guidelines, restriction and prior approval, education	Before-after	No change in LOS, mortality and readmission rates in the pre- and post-intervention periods.
Ng *et al*., 2008, Hong Kong [70]	1800-bed acute hospital	Guideline, antibiotic order form, restriction, review and feedback	Before-after	Significant difference in LOS between pre- and post-ASP (7.46 *vs*.6.97 days, (*p* < 0.001). NS difference in mortality (8.8% *vs*. 8.4%, (*p* = 0.28). Significant unplanned readmissions related to infections post-ASP (17.6% *vs*. 18.7%, (*p* = 0.008).
Chan *et al*., 2011, Taiwan [71]	3500-bed medical center	Hospital-wide computerized antimicrobial approval system linked to electronic medical record, monitoring, review, feedback	Prospective interventional	Decreasing trends in mortality over a period of 7 years 3.45%, 3.53%, 3.41%, 3.30%, 3.28%, 3.27%, and 3.23%.
Liew *et al*., 2012, Singapore [72]	1559-bed tertiary-care hospital	Guidelines, posters, prospective review with intervention	Retrospective review of ASP interventions	Shorter LOS in patients whose physicians accepted interventions than those interventions were rejected (19.9 *vs*. 24.2 days, *p* < 0.001). NS (*p* = 0.191) difference in overall mortality and infection-related mortality between the two groups. Infection-related readmission and 14-day re-infection was higher in patients whose physicians rejected AS interventions (*p* < 0.001 and *p* = 0.009) respectively.
DiazGranados, C., 2012, USA [73]	ICU at a 1000-bed community teaching hospital	Prospective audit with intervention and feedback (PAIF)	Prospective quasi-experimental	NS (*p* = 0.68) difference in mortality between patients in intervention group and baseline. Hospital and ICU LOS was shorter in the PAIF group than the baseline.
Rimawi *et al*., 2013, USA [74]	24-bed medial ICU at 861-bed teaching hospital	Review and feedback	Before-after	Significant reduction in mechanical ventilation days (*p =* 0.0053), LOS (*p =* 0.0188), and hospital mortality (*p* = 0.0367). NS difference in medical ICU mortality (*p =* 0.4970).
Lin *et al*., 2013, Taiwan [75]	415-bed non-university affiliated community teaching hospital	Education, prospective review with intervention and feedback	Before-after	NS difference inLOS and mortality.
Tsukamoto *et al*., 2014, Japan [76]	600-bed university teaching hospital	Daily review and feedback	Before-after	30-day mortality was lower in post-intervention than pre-intervention period (14.3% *vs*. 22.9%, *p* = 0.2).
Pasquale *et al*., 2014, USA [77]	577-bed community teaching hospital	De-escalation, dose optimization, ID consult	Retrospective review of ASP interventions in patients with ABSSSIs	Mean LOS was shorter (4.4 days *vs*. 6.2 days; *p* < 0.001) compared to historical data. 30-day all-cause readmission rate was lower (6.5% *vs*. 16.71%, *p* = 0.05) in intervention group but 30-day ABSSSI readmission rate did not differ between intervention and historical groups (*p* = 0.483).
Rosa, Goldani & dos Santos, 2014, Brazil [78]	Hematology ward of teaching hospital	ASP guidelines for cancer patients with febrile neutropenia	Prospective cohort	Adherence to ASP guidelines was associated with lower mortality (hazard ratio, 0.36; 95% confidence interval, 0.14–0.92).
Lew *et al*., 2015, Singapore [79]	1500-bed teaching hospital	De-escalation of carbapenem therapy	Retrospective review of ASP interventions	NS difference in clinical success, survival at discharge, 30 day mortality, 30 day readmission and LOS between de-escalated and non-de-escalated groups. There was difference in antibiotic-associated diarrhea (4.4% *vs*. 12.5%; *p* = 0.015) the between the two groups.
Okumura, da Silva & Veroneze, 2015, Brazil [80]	550-bed university hospital	Bundled ASP comprisingdaily review and feedback, de-escalation, education, follow up till resolution	Retrospective historical cohort	30-day mortality was lower with bundled ASP (*p* < 0.01) than conventional ASP (which comprised passive chart review, discussion with ID and telephone call when intervention was necessary).

NS—Non-significant, LOS—Length of stay, ABSSSIs—acute bacterial skin and skin structure infections.

## 4. Discussion

Our review identified a number of quality measures used in assessing ASP in primary studies. These include change in antimicrobial use, cost savings, resistance patterns of some difficult to treat organisms, rates of CDI, length of stay (LOS), readmission rate and mortality. These measures were classified into two main categories namely process and outcome measures [20,81]. Change in antimicrobial use (such as total quantity of antimicrobial or targeted antimicrobial class) measured usually in the WHO recommended defined daily dose (DDD)/100 or 1000 patient-days [81] is a process measure [4,20]. Other process measures recommended for use in assessing ASP include documentation of indication for antibiotic prescribed, documentation of stop/review date, 48–72 hours review after initiation of antibiotic therapy, level of adherence to hospital-specific guidelines, level of acceptance of AMS recommendations, time to appropriate therapy in patients with sepsis, and rate of de-escalation of initial therapy [12,14,82]. Outcome measures are categorized into microbiological, clinical and financial outcomes [82]. Microbiological outcomes include measures such as percentage of difficult to treat organisms e.g., MRSA, ESBL-producing *Enterobacteriaceae*, rate of isolation of resistant organisms, and rate of CDI [83]. Clinical outcome measures used in assessing impact of ASP include all-cause mortality, LOS and readmission rates; clinical improvement and rate of adverse antimicrobial reactions have also been recommended [20,82,84].

Currently, there are no standard, universally accepted metrics for assessing ASP. For example, DDD whilst widely used in quantifying and reporting antimicrobial use continues to be debated because of its limitations [85]. The limitations of using DDD include its inability to provide information on the number of patients actually exposed to antibiotics; it cannot be used for children, and it underestimates the use for drugs that require reduced dosage due to renal impairment [85,86]. Morris *et al*. [18], in a structured panel to determine quality metrics for ASP, suggested days of therapy/1000 patient-days as a more appropriate measure for public reporting of ASP impact. Similarly, Aldeyab *et al*. [85] in a study that adjusted DDD to include age-adjusted comorbidity score (DDD/100 bed-days/age-adjusted comorbidity score) concluded that the modified unit provides “an innovative approach to measuring antibiotic use while taking into account the effect of patient case mix”. Prescribed daily dose has also been suggested as an alternative or a complement to DDD [20]. Whether these metrics provide the appropriate standards for assessing ASP has not been determined. However, the majority of the studies that reported significant reductions in antimicrobial use employed DDD/1000 or 100 patient-days as the metric [24,33,34].

Assessing the impact of ASP on resistance using the identified metrics has inherent limitations. This is because several factors affect the development of resistance, which makes it difficult to establish a clear causal association between AMS interventions and decrease in resistance [9,20,49]. However, ASP especially those employing restriction on use of ‘high-risk’ antibiotic classes (second- and third-generation cephalosprins, fluoroquinolones) have been shown to reduce resistance and/or improve bacterial susceptibilities [47,63,65]. Although stewardship interventions have been shown to reduce resistance, their use as a primary measure for evaluating ASP has been cautioned [87]. Rate of CDI has been used as a measure for assessing ASP. Programs incorporating restriction or avoidance of the ‘high-risk’ antibiotic classes and clindamycin are notably associated with significant reduction in CDI rate [42,44,51,52,54,58,63]. Studies with marked reduction in CDI often also have strict infection control programs in place; which makes the association between ASP and the reduction in CDI rate difficult. However, infection control alone has been shown not to effectively control the outbreak of CDI. A significant reduction in rates followed stewardship interventions that involved restriction or avoidance of the ‘high-risk’ antibiotics [42,52,63].

The primary goal of ASP is to optimize patient outcomes. Six out of 13 studies included in this review reported non-significant difference in mortality [69,71,72,73,75,79]. Six studies reported shorter LOS between the pre- and post-intervention periods [68,70,72,73,74,77]. Notably, Ng *et al*. [70] reported significant difference in LOS between the periods before and after ASP implementation (7.46 and 6.97 respectively; *p* < 0.001). Interestingly, the same study reported statistically significant (*p* < 0.001) higher unplanned readmissions related to infections post-ASP.

Evaluation of ASP requires the use of patient-specific measures that demonstrate attainment of the primary goal. However, some limitations affect effective evaluation. These include difficulty in establishing a clear causal association between ASP interventions and measures such as mortality and LOS due to confounders that affect these measures [9,20]. Mortality related to antimicrobial-resistant organisms and infection-related hospital stay has been suggested as better patient measures for use in assessing impact [18,70]. Lack of personnel, funds, and health information technology personnel, and the inability to generate and analyze ASP-specific data have also been identified as limitations to effective ASP [9,88]. Inadequate study design also limits a clear association between ASP interventions and reported impact. Studies of interventions to improve hospital antimicrobial use are reported to be largely of poor design [11]. 

This review did not apply the strict quality criteria required for a systematic review of included studies, and risk of bias was not assessed. The purpose of this review was to provide an overview of the quality measures used in assessing ASP in primary studies, therefore all study designs were included. Studies assessing patient specific outcomes were of particular interest. Future work is planned to include evaluation and impact of ASP in pediatric patients.

## 5. Conclusions

Patient outcomes need to be a key component of ASP evaluation. The choice of metrics is influenced by data and resource availability. Controlling for confounders and unintended adverse consequences must be considered in the design of evaluation studies to adequately capture the impact of ASP. This review provides a starting pointfor compiling standard outcome metrics for assessing ASP.
